# Laparoscopic–Endoscopic “Rendezvous” Procedure in Pediatric Gastrointestinal Surgery—Case Series

**DOI:** 10.3390/children8090770

**Published:** 2021-09-01

**Authors:** Radoica Jokić, Jelena Antić, Svetlana Bukarica, Miloš Pajić, Ivana Fratrić

**Affiliations:** 1 Institute for Child and Youth Healthcare of Vojvodina, Clinic for Pediatric Surgery, Hajduk Veljkova 10, 21000 Novi Sad, Serbia; jelena.antic@mf.uns.ac.rs (J.A.); svetlana.bukarica@mf.uns.ac.rs (S.B.); milos.pajic@mf.uns.ac.rs (M.P.); ivana.fratric@mf.uns.ac.rs (I.F.); 2 Faculty of Medicine, University of Novi Sad, Hajduk Veljkova 3, 21000 Novi Sad, Serbia

**Keywords:** “rendezvous” procedure, laparoscopy, endoscopy, gastrointestinal surgery, polyp

## Abstract

Laparoscopic–endoscopic “rendezvous” procedures were introduced in surgery for common bile duct stone treatment but are now widely used in other fields of abdominal surgery. An endoscopist navigates a surgeon during the same operative procedure and, thus, enables a better visualization of the location, resection margins, bleeding control, less thermal damage, etc. Here, we present case series of 11 patients that were treated using a “rendezvous” procedure for gastrointestinal lesions on different parts of the gastrointestinal tract such as juvenile polyps on the colon (transversum, ascendens, cecum, sigma), leiomyomatosis of the stomach, Peutz–Jeghers intestinal polyposis, hyperplastic gastric polyp, ectopic pancreatic tissue in the stomach, gastric trichobezoar, and gastric schwannoma. “Rendezvous” procedures are suitable for intestinal lesions that could not be resected endoscopically due to their size, morphology and/or location. In our experience this procedure should be used for endoscopically unresectable lesions as it decreases the time of surgery, possibility of iatrogenic injury, bleeding and technical inability. Furthermore, this procedure has been shown to better navigate the surgeon during laparoscopic surgery, especially in treating polyps in particularly difficult locations such as the duodenum or cecum, and it decreases conversion rates. However, conversion is sometimes necessary, in order to assure all oncological principals are respected, and the best option in some cases.

## 1. Introduction

The term “rendezvous” comes from the French word meaning appointment and is used in surgery to point out the procedures in which the surgeon and endoscopist meet each other at the point of the lesion [1]. This procedure was firstly used for common bile duct stone treatment [1] but was introduced in other gastrointestinal (GI) areas as well. In GI surgery, this procedure is used for lesions that, for some reason, could not be resected completely endoscopically [2] or in laparoscopic procedures in which endoscopic navigation is necessary [3]. Endoscopic navigation is used in this technique for accurately determining the surgical field of work. The surgery is then finished laparoscopically or with conversion to standard open surgery.

Although well described in adults, this procedure was introduced in children with symptomatic cholecysto-choledocholithiasis more recently [4] and consists of a laparoscopic cholecystectomy and simultaneous endoscopic retrograde cholangiopancreatography.

A much more common implication of a “rendezvous” procedure is a percutaneous endoscopic gastrostomy (PEG) in children in whom prolonged enteral feeding is required. In this technique, transillumination is used to determine the gastrostomy site. Percutaneous endoscopic gastrostomy is now feasible even in children weighting less than 10 kg according to a recent study [5].

The aim of this study is to present 11 patients that were treated using laparoscopic–endoscopic “rendezvous” procedures for the gastrointestinal lesions on different parts of the GI tract, to highlight the benefits of the laparoscopic–endoscopic “rendezvous” procedure and to address its implications in pediatric gastrointestinal surgery especially in treating polyps in difficult locations such as the stomach, duodenum, jejunum or cecum, and for treating tumors that are suspected for malignancy where the principle of minimal invasiveness should not come before the adequate radical resection.

## 2. Method

This study was designed as retrospective case series study and was conducted according to the guidelines of the Declaration of Helsinki. It was approved by the Institutional Review Board of Institute for Child and Youth Healthcare of Vojvodina (Number 2970–2 from 27 July 2021). The Health Information System and medical source documentation were used to retrieve the data of the patients. Medical documentation of 11 patients in whom a laparoscopic–endoscopic “rendezvous” procedure was performed in the last 13 years was used. Informed consent was obtained from all subjects (parents/guardians) involved in the study.

All subjects underwent endoscopy (esophagogastroduodenoscopy and/or colonoscopy) during which gastrointestinal pathology was confirmed, but endoscopic treatment was not possible at the time. 

One day prior to the surgery, all patients were given Picoprep (picosulfate 10 mg/magnesium oxide 3.5 g/citric acid 12 g) solution according to their weight. This solution was given twice a day (in the morning and in the afternoon). During that day, children were encouraged to drink from 500 mL of clear solution (for children weighting 0–10 kg), 1000 mL (10–20 kg), 1500 mL (20–40 kg) to 2000 mL for children weighting more than 40 kg.

## 3. Case Series Presentation

Here we present eleven patients that were treated using laparoscopic-endoscopic “rendezvous” procedures for the gastrointestinal lesions on different parts of GI tract at our Institute.

Table 1 summarizes demographic characteristics, symptoms, diagnostic procedures, pathohistological finding, necessity for conversion and complications in patients in whom laparoscopic-endoscopic “rendezvous” procedure was performed.

The first patient in our case series is a 10-year-old boy who was examined by a gastroenterologist and a gastroduodenoscopy was performed with a diagnosis of chronic duodenitis, suspected pseudo polyp of the duodenum and inactive chronic atrophic gastritis in the region of the stomach’s antrum. A colonoscopy revealed the mild form of chronic colitis and a polyp in the sigmoid colon with a diameter of 1 cm and another one with a diameter of 3 cm on the long loop in the descending colon. The colonoscopy showed numerous small polyps in the rectum, colon transversum, ascending colon and cecum. The surgical decision for a combined laparo-endoscopic polypectomy was made. Pneumoperitoneum (12 mmHg) was created using the open Hasson technique, through infraumbilical incision and 5-millimeter port. Two 5-millimeter working ports were inserted in the left and right iliac region. An endoscopist navigated a surgeon to the polyps in the descending colon and thus a precise enterotomy was performed. Both polyps were simultaneously exteriorized and excided. The wall of the colon was closed using direct intracorporeal sutures. The rest of the surgery and postoperative period were uneventful.

Another case that we present here is a 3-year-old boy in whom an upper endoscopy and colonoscopy was performed and revealed chronic gastritis and a rectal polyp more than 15 cm from the anocutaneous line. Infraumbilical open access with the 5-millimeter Hasson technique was used to create pneumoperitoneum. Two 5-millimeter working ports were positioned in the right and left iliac region. An intraoperative colonoscopy showed a sessile polyp in the sigma at about 22 cm from the anocutaneous line. Using the navigation of an endoscopist, a surgeon located the site of the incision on the anterior wall of the sigma. This part of the surgery was performed as a combined laparoscopic–endoscopic “rendezvous” procedure. The mobile part of the sigma with the polyp was exteriorized through the slightly enlarged left working port. The polyp was sessile with the blood on its surface and seemed vulnerable. Morphologically, it appeared as a hemangioma. The complete excision of the polyp was performed with a resection of this segment of the colon and consecutively, termino-terminal anastomosis in two layers was performed. AN abdominal drainage was placed, and hemostasis was unremarkable. 

**Figure 1:**
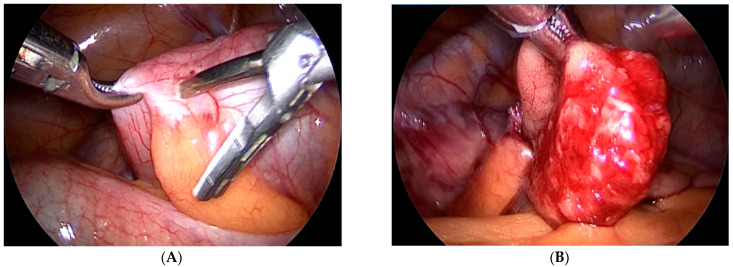
(**A**) Endoscopic guidance from the lumen of the intestine and a place of enterotomy, (**B**) Complete visualization of the polyp and its resection.

Our third case is a 2-year-old child who was admitted to hospital due to blood and mucus in the stool. Intraoperative colonoscopy revealed polypoid form diameter around 2 cm, 30 cm from the anocutaneous line that showed ulcerations and was on the wide base. Biopsies were sent for pathohistological examination. The endoscopic histologic diagnosis was a polyp of the sigma and chronic colitis. A polypectomy was performed using a laparoscopic–endoscopic “rendezvous” procedure. Infraumbilical open access with the 5-millimeter Hasson technique was used to create pneumoperitoneum. The working ports were placed in epigastric and in the upper right quadrant. By lighting from the lumen of the colon, an enterotomy was performed (Figure 1A). The polyp was visualized, resected in its base (Figure 1B), and sent for definitive pathohistological examination. A direct enterorrhaphy during the laparoscopy was performed in the standardized manner. The operation was completed, and the postoperative period was uneventful.

The fourth case is a 16-year-old male patient in whom an esophagogastroduodenoscopy revealed a gastric tumor. An MRI and CT of the abdomen were performed. These diagnostic procedures showed a submucosally localized tumor of the stomach in the region of the cardia/small curve, dimensions 48 × 18 mm. After the preoperative preparation, the patient was scheduled for a combined laparo-endoscopic “rendezvous” procedure. Infraumbilical open access with 5-millimeter Hasson technique was used to create pneumoperitoneum (12 mmHg). The esophagogastroduodenoscopy revealed the tumor just near the cardia of the stomach. An ultrasonic scalpel was used for the gastrotomy of the anterior wall near the esophagogastric junction navigated by an endoscopist, but the tumor could not be identified; therefore, the appropriate conversion was made. An upper median laparotomy was performed, the previous gastrotomy was just slightly enlarged and the submucosal lesion, diameter around 4 cm, was identified by palpation. An incision of the mucosa was performed following the meticulous dissection of the tumor. The tumor was sent for pathohistological examination and the operation was completed in the standardized way, firstly with the reconstruction of the mucosa and with the closure of the gastrotomy in two layers. Two abdominal drains were placed (contact subhepatic and in the Douglas space).

Our fifth case is 8-year-old girl who underwent urgent laparoscopic exploration under general anesthesia during which intestinal invagination was noted. Laparoscopic desinvagination was performed and through one enterotomy, the two polyps that caused the invagination were removed. The small bowel with enterotomy was exteriorized and a gastroscope was inserted through the previously mentioned enterotomy to control the region of invagination. On the fifth postoperative day, clinical symptoms of intestinal obstruction were developed again. A control X-ray confirmed ileus. A diagnostic laparoscopic exploration under general anesthesia was performed again, which verified re-intussusception and extreme intestinal distension. Due to operational findings, the conversion to standard open surgery was made. Through medial laparotomy, the abdominal cavity was entered. Re-invagination was confirmed and manual desinvagination was performed. The additional four enterotomies and polypectomies were performed. Without palpation it was not possible to reveal further polyps, which could have been the cause of new invaginations. Histopathological examination confirmed Peutz–Jeghers polyposis (polypus hamartomatosus mucosae intestine).

**Figure 2:**
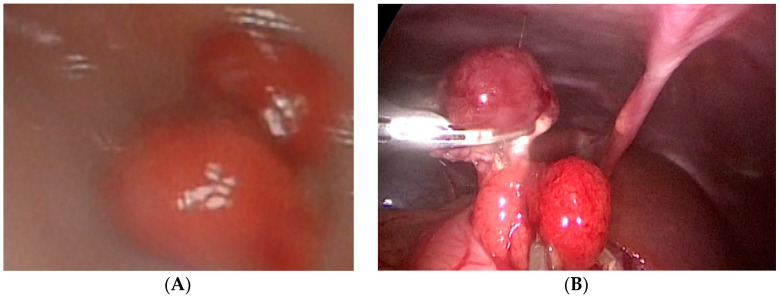
Endoscopic visualization of two polyps and laparoscopic polypectomy. (**A**) After having polyps endoscopically visualized; (**B**) A double polypectomy was made using an ultrasonic knife.

Case number six is a 3-year-old girl who presented with hematemesis and melena. An endoscopy revealed two oval polyps located at the antrum of the stomach with the largest diameters of 1 and 1.5 cm. One of these polyps was bleeding actively and an endoscopic injection hemostasis was conducted. Due to the technical inability, an endoscopic polypectomy was not performed. The next day, a combined laparoscopic–endoscopic “rendezvous” polypectomy was completed. After having polyps endoscopically visualized (Figure 2A), an anterior gastrotomy was performed. A double polypectomy was made using an ultrasonic knife (Figure 2B). The gastric wall was sutured, and the operation was completed with an omentopexy.

**Figure 3:**
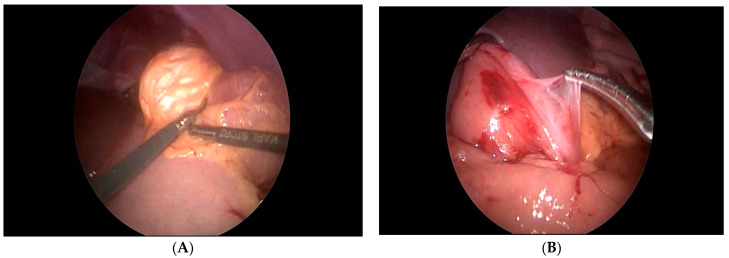
(**A**) Endoscopic navigation by transillumination of the duodenum and jejunum, (**B**) Cattell–Braasch maneuver.

**Figure 4:**
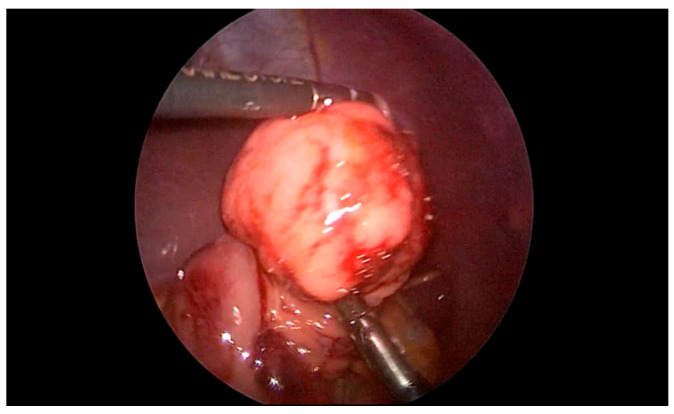
Laparoscopic excision of the duodenal polyp (The straightened duodeno-jejunal junction allowed for the visualization and resection of the duodenal polyp during repeated mucosal mobilization).

The seventh case in our case series is a 6-year-old boy who was referred to our clinic for a suspected ileocecal invagination. A hydrostatic desinvagination guided by ultrasound was attempted, and a patent ileocecal valve was confirmed. A pseudo kidney formation remained, indicating the possibility of an invagination of the duodenum and small intestine. A repeated ultrasound demonstrated that a pseudo-invagination was still present in the upper left quadrant. An MR-enterography was performed and demonstrated a 20-centimeter long suspected intussusception in the left hemiabdomen beginning in the third part of the duodenum and continued below the ligament of Treitz. Multiple polyps of the duodenum and jejunum were suggestive of Peutz–Jeghers syndrome as well. The most proximal polyp with a diameter of 6 cm produced a marked narrowing of the distal part of the duodenum and proximal jejunum. An esophago-gastro-duodenoscopy was performed, and a biopsy was taken. The biopsy confirmed the presence of a hyperplastic polyp of the duodenum. Then, the duodenum and jejunum were exposed using a combined laparoscopic–endoscopic “rendezvous” procedure (Figure 3A). A vertical incision was made on the highest position of the jejunal wall, and the mucosa was mobilized. However, the polyp could not be visualized during the first attempt. Then, we decided to use a partial Cattell–Braasch maneuver and resection of the ligament of Treitz for better mobilization of the duodenal mucosa (Figure 3B). After the resection of the ligament, the natural curvature of the duodeno-jejunal flexure was straightened. The straightened duodeno-jejunal junction allowed for the visualization and resection of the duodenal polyp (Figure 4). A second enterotomy was performed distally on the jejunum as well and two more polyps were resected (diameter 20 and 15 mm, respectively). The histology confirmed Peutz–Jeghers syndrome.

**Figure 5:**
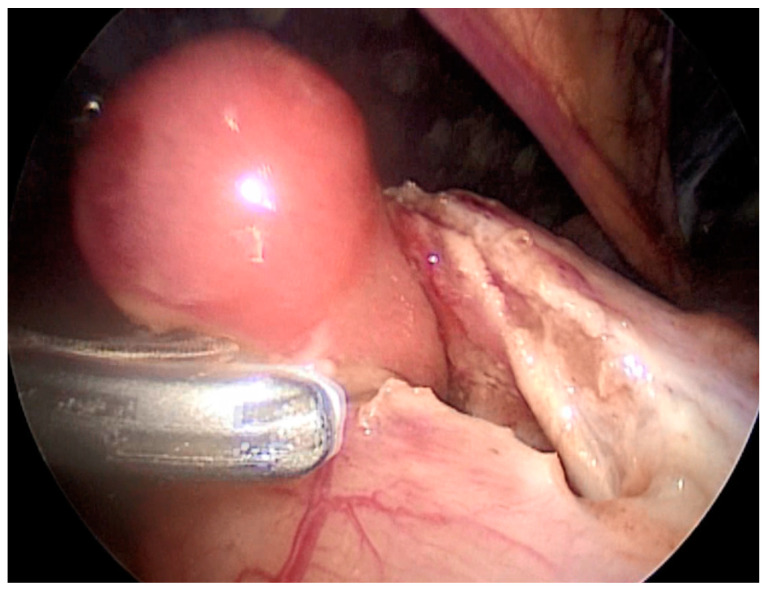
Laparoscopic gastrotomy and polypectomy (pancreatic tissue ectopy in the stomach) under the guidance of endoscopist.

Our eighth case is a 9-year-old boy who was admitted to the clinic for pediatric surgery for the planned polypectomy of the stomach. Gastroduodenoscopy revealed a submucosal polypoid formation with a diameter around 1.5 cm in the region of the pylorus. The child was referred to a pediatric surgeon. Under the guidance of endoscopist, a laparoscopic gastrotomy and polypectomy was performed in the way already described (Figure 5). The polyp was sent for histological evaluation and gastroplasty with omentopexy was performed. The histological evaluation revealed pancreatic tissue ectopy in the stomach.

**Figure 6:**
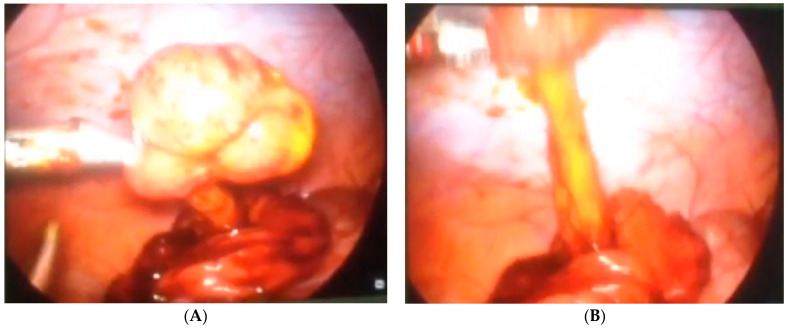
(**A**) Laparoscopic excision of the cecum polyp, (**B**) polyp on the long loop.

**Figure 7:**
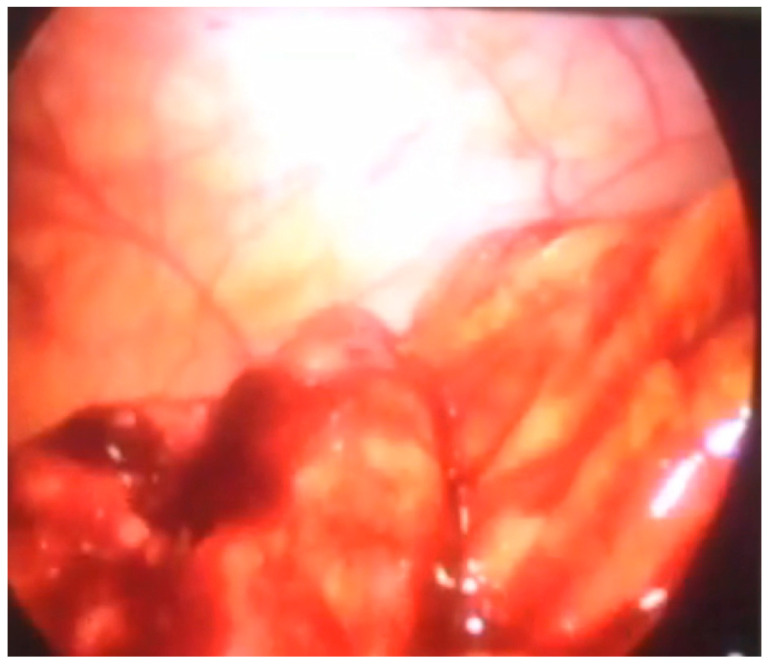
Edematous ileocecal valve that could be missed for polyp.

Another patient was hospitalized at our Institute due to abdominal pain and blood in a stool. A colonoscopy revealed a polyp in the region of the cecum. A simultaneous laparoscopic–endoscopic “rendezvous” polypectomy was performed, and the polyp was sent for histologic examination (Figure 6A). The polyp was on the long loop (Figure 6B) and the main difficulty was to differentiate the polyp from the edematous ileocecal (Bauhin’s) valve (Figure 7). The enterotomy on the cecum was sutured with direct intracorporeal sutures in two layers. The histology confirmed the diagnosis of a juvenile polyp.

## 4. Discussion

The tendency of using minimally invasive techniques as diagnostic and therapeutic tools has led to a wide use of endoscopy in diagnosing and treating some specific GI lesions [7]. Despite having great results, this technique carries significant risks if performed in excess in cases of tumors that are greater than 2 cm or tumors that arise from muscularis propria or deeper layers. Furthermore, thermal damage from the excessive use of an endoscopic electrocautery can cause later perforation and/or bleeding [8]. The new literature findings show that experienced endoscopists can perform a resection of gastric tumors arising from muscularis propria or deeper layers without such complications [9]. Achieving a complete resection remains a high task when trying to remove these tumors endoscopically. This was the reason to combine endoscopic and laparoscopic approaches in order to better localize the tumor, achieve a complete resection with adequate margins and control the surgical results from the inside and outside of the GI tract [10]. All this is merged in “rendezvous” procedures where laparoscopic resections are used with the guidance of the intraluminal endoscopic observations. In our five reported cases (Case number 4, 6, 8, 10, 11), the pathology of the stomach was the main reason for the laparoscopic endoscopic “rendezvous” procedure. In all these cases, after having polyps endoscopically visualized, an anterior gastrotomy was performed, a polypectomy was made using an ultrasonic knife, following the gastric wall suture with omentopexy at the end of the procedure. In most cases, operations were completed laparoscopically, but in some cases (with respect to oncological principals, technical or other inability) conversion into the standard open technique was necessary.

When treating gastric lesions, pediatric surgeons are usually faced with gastric polyps (case number 6 and case number 10), but sometimes leiomyoma (case number four), gastric schwannoma (case number 11), GIST or ectopic pancreatic tissue (case number eight) can be found. Although benign tumors are predominately in the pediatric age group, malignant tumors such as GIST can be found as well. One of the pathologies treated with the laparoscopic–endoscopic “rendezvous” procedure at our Institute is gastric schwannoma, which is a benign tumor, but still with the possibility of recurrence; therefore, all the above-mentioned pathologies should be monitored and followed in the adequate time period.

The incidence of ectopic pancreatic tissue, heterotopic pancreas or pancreatic rest varies from 2 to 15% according to autopsy reports or 0.2 to 5% according to surgical reports [11,12]. They could be symptomatic or without any symptoms. The most common site is the stomach and duodenum [12], but Italian researchers reported most of the cases in the pediatric population in the Meckel diverticulum and small bowel distal to duodeno-jejunal junction [11]. Here, we report the case of heterotopic pancreatic tissue in the stomach (Case number eight).

One of the main disadvantages of minimally invasive surgery is the lack of the palpation method that could be replaced with 3D technology but is still one of the reasons for conversion. The other way of overcoming this disadvantage is a video assisted type of operation that implies exteriorization of the mobile structure and continuation of the operation extracorporeally with all the benefits of the standard open surgery. This was used in the case of the gastric schwannoma. Due to the submucosal position of the tumor, it was hard to define the tumor margins macroscopically and that is why an intraoperative palpation had to be used. Furthermore, this manual palpation was performed in cases number two and five.

Gastric lesions less than 2 cm in diameter found during an endoscopy are usually resected during the same procedure. However, the location of the lesion (near the esophagogastric junction, pylorus or on the posterior gastric wall) and the layer of origin (involving muscularis propria) can sometimes disable the endoscopist to finish the resection successfully [10,13,14]. The experience of the endoscopist and the availability of the necessary tools are sometimes lacking and that is why an alternative way had to be found.

To address this issue, the combined laparoscopic–endoscopic “rendezvous” procedure was promoted decades ago and has been successfully implementing since then. We found out about this procedure 14 years ago from a paper by G. Pelizzo and J. Schleef and decided to implement it at our Institute (as reported in Case number 6, in a child with two gastric polyps, one of which was bleeding actively) [15]. Our first successful “rendezvous” procedure was performed to resect gastric polyps. The initial experience on resecting a gastric polyp using this technique enforced us to expand the field of its implementation to pathologies in gastric tumors, duodenum, jejunum and ileum using an upper endoscopy in all these procedures. For treating lesions on the colon and sigma, we are using a colonoscopy guided “rendezvous” technique, as described in the first three cases in this report.

For decades after minimally invasive laparoscopic surgery has been introduced, duodenal lesions were impossible to resect using this approach. Recently, more and more surgeons are attempting to extend their field of work to the duodenum and endoscopic guidance can be of tremendous help during this procedure. Here, we reported a case of a duodenal polyp treated in this way. The Cattell–Braasch maneuver and resection of the ligament of Treitz were used in order to straighten the natural duodeno-jejunal flexure. In 1960, Cattel and Braasch described the technique for the mobilization of the third and fourth portion of the duodenum that is used until today [16]. This maneuver consists of dissection of the line of Toldt from the common bile duct until the ligament of Treitz. Additional mobilization of the suspensory muscle of the duodenum (ligament of Treitz) was performed in the case we report here for complete mobilization of the duodenum. After the resection of the ligament, the natural curvature of the duodeno-jejunal flexure was straightened. The straightened duodeno-jejunal junction allowed for the visualization and resection of the duodenal polyp during repeated mucosal mobilization. Nowadays, laparoscopic endoscopic cooperative surgery, combining endoscopic submucosal dissection and laparoscopic seromuscular incision, is reported to be successful in treating duodenal lesions as well [17].

Combined laparoscopic–endoscopic procedures are sometimes used in treating lesions in the small intestine such as polyps and others. Here, we reported two cases of Peutz–Jeghers Syndrome, a condition that is inherited in autosomal dominant manner and effects one in 25,000–300,000 newborn babies [18]. In a paper by Pelizzo et al., a patient with Peutz–Jeghers Syndrome was treated with a “rendezvous” procedure, but the lesions were identified with the colonoscope inserted through the mouth of the patient. In our version of this procedure, we exteriorized the bowel through an enlarged incision of one port and used a gastroscope to examine the lumen of the small intestine proximal and distal from the laparoscopically created enterotomy.

A laparoscopic approach to treat polyps of the cecum is also rare in the literature [19]. Giavarini et al. [20] reported 15 patients with a median polyp size of 3 cm localized in the anterior, posterior and lateral wall of the cecum that were resected using a colonoscopy-assisted laparoscopic wedge resection (CAL-WR) with the success rate of 100%. This procedure with little modification is reported by Leicher et al. [19] for “difficult” polyps in terms of location—cecum, hepatic flexure, splenic flexure and sigmoid colon. Polyps of the cecum are considered and described in the literature as “difficult” because of their location and the proximity of the ileocecal valve [3]. These lesions were usually treated by open surgery, but nowadays, with the introduction of endoscopically assisted laparoscopic procedures, many of these lesions could be resected laparoscopically, as we described here. Our patient in whom a cecum polyp was identified, as reported in Case number nine, had no reported previous appendectomy; therefore, the possibility of a “stump” left after the appendectomy was rejected. The possibility of misdiagnosing the edematous ileocecal valve as a cecal mass is well documented since 1955 in terms of ileocecal prolapse [21], lateral position [22] or hypertrophy of the valve [23]. However, with the advancement of radiology and colonoscopy, misdiagnosing the ileocecal valve as a polyp is now minimal. The efficacy of every colonoscopy is now measured as a demonstration of the ileocecal valve and the detection of the polyps [24].

With the help of a colonoscopy and guidance to the pathologic lesion, a surgeon can make more a precise incision at the tenia line. This makes the localization of the lesion in the colon and sigma easier, as in our first three presented cases and in case number nine, and then the operation can be finished laparoscopically without conversion. The polyp of the sigma reported in case number two was sessile, which further supported our decision for a “rendezvous” procedure, as it would be difficult to localize it without the help of an endoscopist.

An early postoperative complication in our case series was reported in case number three. The analysis of the operative wound swab revealed Staphylococcus aureus on the seventh postoperative day. Generally, during an operation on the colon, the possibility of a postoperative surgical site infection and general infection is significant [25]. We were aware of this possible complication before the operation. Numerous colon preparation measures are in use worldwide to lower the incidence of infection. Our patient was treated with antibiotics together with local treatment of the surgical site infection and this complication resolved quickly. Initially, we once treated the polyp of the colon with resection, but an excision of the polyp is more appropriate and now this is our standard practice, which is less invasive but carries some risk of surgical site infection.

Combined laparoscopic–endoscopic “rendezvous” procedures in gastrointestinal pediatric surgery have a great list of known benefits including less pain, shortened period of recovery, better immune response and earlier discharge. This procedure has proven to be feasible in our Institute and we believe that it should be promoted to general adult surgery as well.

After the initial experience of our center with the laparoscopic–endoscopic “rendezvous” procedure, we came across some technical challenges, such as the inability of reaching gastrointestinal lesions with the available gastroscope or colonoscope. In our opinion, a device similar to the one used for an esophagogastroduodenoscopy, but longer, would be of great help for treating similar lesions in the future.

Another limitation of this procedure is time. Although less invasive with a shortened postoperative stay in the hospital, it requires longer time in the operating room with well-trained personnel. Despite all the efforts of the surgeon and gastroenterologist, sometimes conversion to standard open surgery is necessary. The necessity of the gastroenterologist and trained personnel for endoscopic procedures in the OR is another technical challenge that could be easily resolved by adequate communication and planning the procedure.

## 5. Conclusions

Although endoscopy is the “gold standard” for treating pathological lesions of the gastrointestinal tract, the benefits of the laparoscopic–endoscopic “rendezvous” procedure in treating polyps show a high level of application, especially in difficult locations such as the duodenum or cecum and for treating tumors of the GI tract that are suspected of malignancy where the principal of minimal invasiveness should not come before the adequate radical resection. If necessary, conversion should be performed as well in order to assure all the oncological principals are respected.

A new innovative endoscope with enough length to reach the small intestine would be of great benefit for the future of the laparoscopic–endoscopic “rendezvous” procedure.

## Figures and Tables

**Table 1:** Demographic characteristics, symptoms, diagnostic procedures, pathohistological finding, necessity for conversion and complications in patients in whom laparoscopic–endoscopic “rendezvous” procedure was performed.

	Age (Years)	Sex	First Symptoms	Indication for the Procedure	Time of the Procedure (from the First Symptoms)	Histological Finding	Conversion	Complications	Comments
1.	10	Male	Blood in the Stool, Watery Stools	* One Polyp (1 cm) in the Sigmoid Colon and One (3 cm) in the Descending Colon	5 Months	Juvenile Polyps	No	No	
2.	3	Male	Blood in the Stool	* Sessile Polyp in the Sigmoid Colon at about 22 cm from the Anocutaneous Line	2 Weeks	Chronic Follicular Colitis and Pseudo Polyp of the Large Intestine	Yes	No	
3.	2	Male	Blood and Mucus in the Stool	* Polyp with wide base and ulceration in the Sigmoid Colon on the 30 cm from the Anocutaneous Line	4 Months	Juvenile Polyp of the Large Bowel with Erosion of Epithelium	No	Surgical Site Infection (Staphylococcus aureus)	
4.	16	Male	Diarrhea with the Blood and Mucus in the Stool	* Gastric Tumor	5 Months	Leiomyoma of the Stomach	Yes	No	
5.	8	Female	Blood in the Stool, Hypochromic Anemia	Ileocecal Intussusception	Immediate	Peutz–Jeghers Polyposis	Yes	Ileus Caused by Re-Intussusception	During Second Operation Additional Four Polyps Were Removed
6.	3	Female	Hematemesis and Melena, Abdominal Pain, Exhaustion and Loss of Appetite	* Two Oval Polyps in the Antrum of the Stomach, One of which was Bleeding Actively	One Day	Hyperplastic Gastric Polyp	No	No	
7.	6	Male	Intermittent Abdominal Colic	Hyperplastic Polyp (6 cm) of the Duodenum and Multiple Polyps in the Jejunum that Caused Invagination in the Left Hemiabdomen (Seen on MR Enterography)	4 Days	Peutz–Jeghers Syndrome	No	No	Partial Cattell–Braasch Maneuver and Resection of Ligament of Treitz was used to Straighten the Natural Curvature of the Duodeno-Jejunal Flexure
8.	9	Male	Abdominal Pain, Nausea, Loss of Appetite, Vomiting	* Submucosal Polypoid Formation Diameter 1.5 cm in the Region of Pylorus	Four Years	Pancreatic Tissue Ectopy in the Stomach	No	No	
9.	10	Male	Abdominal Pain and Blood in the Stool	* Polyp in the Cecum	Three Months	Juvenile Polyp	No	No	Polyp was on the Long Loop and the Main Difficulty was to Differentiate the Polyp from the Edematous Ileocecal Valve
10.	15	Female	Abdominal Pain, Non-bilious Vomiting of Undigested Food and Sometimes Hair	* Trichobezoar and Oval Polyp in the Prepyloric Area of the Antrum	7 Days	Hyperplastic Gastric Polyp	Yes	No	Reference [6]
11.	1	Male	Fever of the Unknown Origin, Recurrent Hematemesis	* Polypoid, Submucosal Tumor Mass 4.8 × 4.0 × 4.0 cm in the Antropyloric Region	6 Weeks	Gastric Schwannoma	Yes	No	Article Sent for Evaluation in Serbian Archive of Medicine

Endoscopic Finding *.

## Data Availability

All source data included in this paper are available at the Institute for Child and Youth Healthcare of Vojvodina.

## References

[1] Borzellino G., Saladino E., Lombardo F., Cordiano C. (2008). Rendez-vous Technique. Biliary Lithiasis.

[2] Menon L., Buscaglia J.M. (2013). Endoscopic approach to subepithelial lesions. Ther. Adv. Gastroenterol..

[3] Liu Z.-H., Jiang L., Chan F.S.-Y., Li M.K.-W., Fan J.K.-M. (2020). Combined endo-laparoscopic surgery for difficult benign colorectal polyps. J. Gastrointest. Oncol..

[4] Rancan A., Andreetta M., Gaio P., Cananzi M., Rossoni R., La Pergola E., Leon F.F., Gamba P. (2019). “Rendezvous” Procedure in Children with Cholecysto-Choledocholithiasis. J. Laparoendosc. Adv. Surg. Tech..

[5] Bawazir O. (2020). Percutaneous endoscopic gastrostomy in children less than 10 kilograms: A comparative study. Saudi J. Gastroenterol..

[6] Bukarica S., Jokic R., Antic J., Stojsic M., Komarcevic A., Fratric I. (2017). Simultaneous combined laparoscopic-endoscopic removal of a large gastric trichobezoar and gastric polypectomy. Srp. Arh. Celok. Lek..

[7] Faulx A.L., Kothari S., Acosta R.D., Agrawal D., Bruining D.H., Chandrasekhara V., Eloubeidi M.A., Fanelli R.D., Gurudu S.R., Khashab M.A. (2017). The role of endoscopy in subepithelial lesions of the GI tract. Gastrointest. Endosc..

[8] Godat S., Robert M., Caillol F., Bories E., Pesenti C., De Cassan C., Ratone J.P., Poizat F., Giovannini M. (2016). Efficiency and safety of endoscopic resection in the management of subepithelial lesions of the stomach. United Eur. Gastroenterol. J..

[9] Westmoreland T., Williams P.W., Brown K.B., Sawaya D.E., Nowicki M.J. (2013). Endoscopic-assisted laparoscopic surgical removal of a gastric neurofibroma in a child. J. Pediatr. Surg. Case Rep..

[10] Hiki N., Nunobe S. (2019). Laparoscopic endoscopic cooperative surgery (LECS) for the gastrointestinal tract: Updated indications. Ann. Gastroenterol. Surg..

[11] Persano G., Cantone N., Pani E., Ciardini E., Noccioli B. (2019). Heterotopic pancreas in the gastrointestinal tract in children: A single-center experience and a review of the literature. Ital. J. Pediatr..

[12] Leung G., Mills J., Bucobo J.C., Docimo S. (2020). Evaluation and management of a pancreatic rest noted during pre-bariatric surgery screening endoscopy. Surg. Endosc..

[13] Aguayo W.G., Rojas C.L., Molina G.A., Cárdenas B.A., Parreño E.F., Melendez S.D., Alvarez M.P., Basantes V.M., Aguayo J.J., Gualotuña F.V. (2021). A hybrid approach for GISTs near the esophagogastric junction, a case report. Ann. Med. Surg..

[14] Hiki N., Nunobe S., Matsuda T., Hirasawa T., Yamamoto Y., Yamaguchi T. (2014). Laparoscopic endoscopic cooperative surgery. Dig. Endosc..

[15] Pelizzo G., Martelossi S., Popoiu M.C., Schleef J. (2007). Laparoendoscopically Assisted Endoscopic Small Bowel Polypectomy in A Patient with Peutz-Jeghers Syndrome. J. Laparoendosc. Adv. Surg. Tech..

[16] Heo Y., Kim D.H. (2020). Medial visceral rotations: The Cattell-Braasch vs. the Mattox maneuvers. Trauma Image Proced..

[17] Ichikawa D., Komatsu S., Dohi O., Naito Y., Kosuga T., Kamada K., Okamoto K., Itoh Y., Otsuji E. (2016). Laparoscopic and endoscopic co-operative surgery for non-ampullary duodenal tumors. World J. Gastroenterol..

[18] (2021). Online Mendelian Inheritance in Man (OMIM) Peutz-Jeghers Syndrome. http://omim.org.

[19] Lecher L.W., Cappel W.H.D.V.T.N., van Westreenen H.L. (2017). Limited Endoscopic-Assisted Wedge Resection for Excision of Colon Polyps. Dis. Colon Rectum.

[20] Giavarini L., Boni L., Cortellezzi C.C., Segato S., Cassinotti E., Rausei S., Dionigi G., Rovera F., Marzorati A., Spampatti S. (2013). Laparoscopic caecal wedge resection with intraoperative endoscopic assistance. Int. J. Surg..

[21] Perkel L.L., Troast L. (1955). Ileocecal valve prolapse simulating cecal polyp. Am. J. Gastroenterol..

[22] Weaver G.A., Davis J.S. (1980). Lateral ileocecal valve presenting as a pedunculated cecal mass and defined by colonoscopically aided air-contrast radiography. Gastrointest. Endosc..

[23] Vanlierde M., Kahn D. (1987). Hypertrophic ileocecal valve simulating a malignant tumour. A case report. S. Afr. Med. J..

[24] Kozan R., Yilmaz T.U., Bastugral U., Kerimoglu U., Yavuz Y. (2018). Factors affecting successful colonoscopy procedures: Single-center experience. Turk. J. Surg..

[25] Martin D., Hübner M., Moulin E., Pache B., Clerc D., Hahnloser D., Demartines N., Grass F. (2018). Timing, diagnosis, and treatment of surgical site infections after colonic surgery: Prospective surveillance of 1263 patients. J. Hosp. Infect..

